# Chemokine Receptor 2 (*CXCR2*) Gene Variants and Their Association with Periodontal Bacteria in Patients with Chronic Periodontitis

**DOI:** 10.1155/2019/2061868

**Published:** 2019-02-04

**Authors:** Denisa Kavrikova, Petra Borilova Linhartova, Svetlana Lucanova, Hana Poskerova, Antonin Fassmann, Lydie Izakovicova Holla

**Affiliations:** ^1^Clinic of Stomatology, Institution Shared with St. Anne's Faculty Hospital, Faculty of Medicine, Masaryk University, Pekarska 664/53, 60200 Brno, Czech Republic; ^2^Department of Pathophysiology, Faculty of Medicine, Masaryk University, Kamenice 5, 62500 Brno, Czech Republic

## Abstract

Periodontitis, an inflammatory disease caused by subgingival Gram-negative (G-) bacteria, is linked with loss of the connective tissue and destruction of the alveolar bone. In the regulation of inflammatory response, chemokine receptor 2 (CXCR2), a specific receptor for interleukin-8 and neutrophil chemoattractant, plays an important role. The first aim of this study was to investigate the *CXCR2* gene variability in chronic periodontitis (CP) patients and healthy nonperiodontitis controls in the Czech population. The second aim was to find a relation between *CXCR2* gene variants and the presence of periodontal bacteria. A total of 500 unrelated subjects participated in this case-control study. 329 CP patients and 171 healthy nonperiodontitis controls were analyzed using polymerase chain reaction techniques for three single-nucleotide polymorphisms (SNPs): +785C/T (rs2230054), +1208T/C (rs1126579), and +1440A/G (rs1126580). A DNA microarray detection kit was used for the investigation of the subgingival bacterial colonization, in a subgroup of CP subjects (*N* = 162). No significant differences in allele, genotype, haplotype, or haplogenotype frequencies of *CXCR2* gene variants between patients with CP and healthy controls (*P* > 0.05) were determined. Nevertheless, *Aggregatibacter actinomycetemcomitans* was detected more frequently in men positive for the C allele of the *CXCR2* +785C/T polymorphism (61.8% vs. 41.1%, *P* < 0.05; OR = 2.31, 95% CI = 1.03-5.20) and for the T allele of the *CXCR2* +1208C/T variant (61.8% vs. 38.9%, *P* < 0.05; OR = 2.54, 95% CI = 1.13-5.71). In contrast, no statistically significant associations of *CXCR2* variants with seven selected periodontal bacteria were found in women. Although none of the investigated SNPs in the *CXCR2* gene was associated with CP, the *CXCR2* gene variants can be associated with subgingival colonization of G- bacteria in men with CP in the Czech population.

## 1. Introduction

Periodontitis is a multifactorial disease that is primarily caused by specific pathogen-associated molecular patterns (PAMPs) and bacterial virulence factors; they trigger an inflammatory host response which results in periodontal tissue destruction and loss of teeth [1, 2]. Chronic periodontitis (CP), the most common form of periodontitis in adults, is either localized or generalized, based on the number of affected sites. The destruction corresponds to the presence of local factors, with a slow-to-moderate rate of progression, but may have periods of rapid progression [3]. CP is strongly associated with “red complex” Gram-negative (G-) bacteria, including *Tannerella forsythia*, *Porphyromonas gingivalis*, and *Treponema denticola* [1, 4]. Although *Aggregatibacter actinomycetemcomitans* is supposed to be the main etiological agent of the aggressive form of periodontitis [5], this bacterium is also connected with CP and some nonoral infections [6].

The host response to anaerobic G- bacteria and their products is an important determinant for progression of periodontal disease. There are a few major risk factors, such as genetic predispositions, systemic diseases, or smoking, which affect the microbial composition in the oral cavity [7, 8]. Cytokines, mediators of host defense and also of periodontal tissue destruction, are considered to be important molecules in the etiopathogenesis of periodontal diseases [9].

Interleukin-8 (IL-8, CXCL8) is known as neutrophil-activating protein-1 (NAP-1) [10, 11]. The effect of IL-8 is mediated by its two receptors—7 transmembrane class A (rhodopsin-like) G protein-coupled receptors (7-TM-GPCRs), so called CXCR1 and CXCR2 [12, 13]. CXCR1 and CXCR2 are expressed on a wide range of leukocytes, including neutrophils, mast cells, and also oral epithelial cells [14, 15]. They are involved in the multiple biological activities, such as initiation and amplification of acute inflammatory reaction, as well as tumor growth, angiogenesis, and metastasis [16–18]. Experimental data suggest that IL-8 and its receptors participate in the elimination of pathogens [19]. A study by Zenobia et al. shows that the recruitment of neutrophils to gingival tissue does not require commensal bacterial colonization but is entirely dependent on *CXCR2* expression [20].

Only a few studies have investigated the variability in *CXCR1* or *CXCR2* genes in relation to CP [21–23], especially in the Brazilian population; however, only the *CXCR2* genotypes and haplotypes have been associated with CP [21]. Based on our previous investigation of *IL-8* gene variability and its association with periodontal bacteria in patients with CP [24], we assumed the role of IL-8 receptor in the etiopathogenesis of periodontal disease.

The first aim of our study was to analyze three SNPs in the *CXCR2* gene +785C/T (rs2230054), +1208T/C (rs1126579), and +1440G/A (rs1126580) in CP patients and healthy nonperiodontitis controls in the Czech population; the second aim was to associate these SNPs with the presence of seven periodontal bacteria in subjects with CP.

## 2. Materials and Methods

### 2.1. Subjects

This case-control association study comprised 500 unrelated Caucasian subjects of exclusively Czech ethnicity from the South Moravian Region. Subjects with CP (number of subjects, *N* = 329) were recruited from the Periodontology Department, Clinic of Stomatology, St. Anne's Faculty Hospital, Brno, over the period of 2013–2018. Healthy nonperiodontitis controls (*N* = 171) were selected from patients who had been referred to the Clinic of Stomatology for reasons other than periodontal disease (such as preventive dental check-ups, dental decay, and orthodontic consultations) during the same period as CP patients, and they were of similar age, gender, and smoking status. Similarly, like the patients, all controls were in good systemic health and had minimally 20 remaining teeth. The exclusion criteria included the presence of diabetes mellitus, cardiovascular disorders (such as hypertension or coronary artery diseases), immunodeficiency, current pregnancy or lactation, malignant diseases, immunosuppression due to medication or concurrent illness, the use of anti-inflammatory drugs or antibiotics during a six-week recruitment period, and the inability to consent [25].

Clinical diagnosis of nonperiodontitis/periodontitis was based on a thorough examination (PI = plaque index, GI = gingival index, etc.), medical/dental history, tooth mobility, and radiographic evaluation. Probing depth (PD) and attachment loss (AL) were collected with a UNC-15 periodontal probe from six sites on every tooth present. The loss of the alveolar bone was determined radiographically, and the decrease in alveolar bone levels was assessed with the Mühlemann index [24]. All participants, no matter whether they agreed or declined to participate or were excluded from the study, were offered periodontitis treatment. The patients were firstly examined by a periodontist, and they had not received scaling and/or root planing minimally six months before measuring periodontal indices [25].

According to their smoking history, the subjects were split into the following groups: nonsmokers (subjects who never smoked) and smokers (former smokers for ≥5 pack-years or current smokers). The pack-years were calculated by multiplying the number of years of smoking by the average number of cigarette packs smoked per day [23]. The demographic data of the studied subjects are shown in Table 1.

### 2.2. Genetic Analysis

Genomic DNA was isolated from peripheral blood by a standard protocol. It was archived in the DNA bank at the Department of Pathological Physiology, Faculty of Medicine, Masaryk University, Brno, Czech Republic. Three SNPs in the *CXCR2* gene (+785C/T (rs2230054), +1208C/T (rs1126579), and +1440A/G (rs1126580)) were analyzed.

For detection of SNP in the *CXCR2* gene at position +785C/T (rs2230054), the original restriction fragment length polymorphism (RFLP-PCR) method with mismatch primers was introduced. Primers were designed by the Primer3Output program. PCR was carried out in a volume of 25 *μ*L containing 100 ng of genomic DNA, 0.5 *μ*M of each primer (Fwd: 5′-TCGTCCTCATCTTCCCGCT and Rev: 5′-GGAGTCCATGGCGAAACTTC), 4 U of *Taq* DNA polymerase (Thermo Scientific, Waltham, USA), 2 mM of MgCl_2_, MgCl_2_-free reaction buffer with NH_4_SO_3_ (Thermo Scientific, Waltham, USA), and 0.5 mM deoxyribonucleoside triphosphate mix (Thermo Scientific, Waltham, USA). Denaturation for 5 min at 95°C was followed by 35 cycles of 95°C for 1 min, 58°C for 1 min, and 72°C for 1 min. The last synthesis step was extended to 7 min at 72°C. The restriction of the PCR product (210 bp) was performed in a volume of 25 *μ*L consisting of 15 *μ*L of the PCR product, 10x CutSmart Buffer, and 4 U of *Bsr*BI enzyme (New England Biolabs, Hitchin, United Kingdom), and incubation was done overnight at 37°C. The length of products after restriction digestion was 210 bp for the TT genotype, 210 bp + 193 bp + 17 bp for CT, and 193 bp + 17 bp for CC. The fragments were visualized by 3.0% agarose gel electrophoresis by ethidium bromide. Sizing of the product was performed using a GeneRuler™ 50 bp DNA Ladder (Thermo Scientific, Waltham, USA).

SNP +1208C/T (rs1126579) in the *CXCR2* gene was genotyped using the 5′ nuclease TaqMan® assay C_8841198_10 for allelic discrimination according to the manufacturer's instructions (Life Technologies, Grand Island, NY, USA). Allele genotyping from fluorescence measurements was then obtained using the ABI PRISM 7000 Sequence Detection System. SDS version 1.2.3 software was used to analyze real-time and endpoint fluorescence data.

The +1440A/G (rs1126580) SNP was genotyped by allele-specific PCR analysis according to the previously published method [26], with a slight modification. A set of appropriate sequences of allele-specific primers and control primers was used: for the allele-specific DNA fragment (Fwd: 5′-AGGCTGGCCAACGGGG/A and Rev: 5′-TCATAGCAGCTTATTCACAAGAC) and for the control DNA fragment (Fwd: 5′-TGCCAAGTGGAGCACCCAA and Rev: 5′-GCATCTTGCTCTGTGCAGAT). There is a difference between the sequences of the allele-specific primers used in our study and those in the work of Renzoni et al. [26]. The length of amplified DNA fragments was also different. The presence of an allele-specific band (435 bp) of the expected size in conjunction with a control band (796 bp) was considered to be positive evidence for each particular allele. The absence of an allele-specific band and the presence of a control band were considered to be a negative indication for a particular allele. Briefly, PCR was carried out in a volume of 25 *μ*L containing 100 ng of genomic DNA, 0.5 *μ*M of each allele-specific primer, 0.4 *μ*M of each control primer, 2.5 U of *Taq* DNA polymerase (Thermo Scientific, Waltham, USA), 2 mM of MgCl_2_, MgCl_2_-free reaction buffer with NH_4_SO_3_ (Thermo Scientific, Waltham, USA), and 0.5 mM deoxyribonucleoside triphosphate mix (Thermo Scientific, Waltham, USA). Denaturation for 5 min at 95°C was followed by 35 cycles of 95°C for 1 min, 62°C for 1 min, and 72°C for 1 min. The last synthesis step was extended to 7 min at 72°C. The fragments were visualized by 2.0% agarose gel electrophoresis by ethidium bromide. Sizing of the product was performed using a GeneRuler™ 50 bp DNA Ladder (Thermo Scientific, Waltham, USA).

### 2.3. Microbial Analysis

The analyses of seven selected periodontal bacteria based on a DNA microarray detection kit (Protean Ltd., Ceske Budejovice, Czech Republic) have been described previously [24, 27]. The presence of bacterial colonization (*A. actinomycetemcomitans*, *T. forsythia*, *P. gingivalis*, *T. denticola*, *Parvimonas micra*, *Prevotella intermedia*, and *Fusobacterium nucleatum*) in subgingival pockets was examined in a subgroup of 162 CP patients before subgingival scaling. Bacterial load was assessed semiquantitatively: (-) undetected, corresponding to a number of bacteria less than 10^3^; (+) slightly positive, which corresponds to a number of bacteria from 10^3^ to 10^4^; (++) positive, corresponding to a number of bacteria from 10^4^ to 10^5^; and (+++) strongly positive, corresponding to a number of bacteria exceeding 10^5^ [28]. The diagnosis of the specific bacterial infection was considered positive when the number of bacterial cells surpassed 10^3^ [23].

### 2.4. Statistical Analysis

Standard descriptive statistics were applied: mean with standard deviations (SD) or median with quartiles for quantitative variables and absolute and relative frequencies for categorical variables. One-way analysis of variance (ANOVA) or Kruskal-Wallis ANOVA was performed to compare continuous variables among the groups. The allele frequencies were calculated from the observed numbers of genotypes. The differences in the allele frequencies were compared by the Fisher exact test, and genotype/haplogenotype frequencies and Hardy-Weinberg equilibrium (HWE) were tested by the *χ*^2^ test. To examine the linkage disequilibrium (LD) between polymorphisms, pairwise LD coefficients (*D*′) and haplotype frequencies were calculated using the SNP Analyzer 2 program (http://snp.istech.info/istech/board/login_form.jsp). Odds ratio (OR), confidence intervals (CI), and *P* values were calculated. *P* values less than 0.05 were considered statistically significant. Where appropriate, the Bonferroni correction was used to adjust the level according to the number of independent comparisons to the overall value of 0.05. The adjusted *P* values are denoted as *P*_corr_. Power analysis was performed with respect to the case-control design of the study, taking the incidence rate of markers. Statistical analysis was performed using the statistical package Statistica v. 13 (StatSoft Inc., USA).

## 3. Results

### 3.1. Case-Control Study

Our population sample consisted of 232 males and 268 females (CP patients 45.6%/54.4%, controls 48.0%/52.0%). 26.3% of CP patients and similarly 27.2% of healthy nonperiodontitis controls were smokers (*P* > 0.05). No significant differences in means of the body mass index (BMI) between CP patients and controls (*P* > 0.05, mean ± SD: 25.45 ± 3.61 kg m^−2^ vs. 26.20 ± 3.77 kg m^−2^, respectively) were detected. Groups of cases and controls were different according to PD, AL, PI, and GI (*P* < 0.01); in CP patients, all mean values were higher than those in controls. The demographic data of the studied subjects are given in Table 1.

The sample size of the study was optimized to reach relevant detectable effect size keeping the standard level of statistical errors, i.e., type I error 0.05 and type II error 0.20 or power of the test 0.80, respectively. The power calculation was optimized on the basis of the Fisher exact and goodness-of-fit tests as statistical tools used in comparing the principal endpoints in the study. Regarding the background relative frequency of the examined phenomenon as 50%, the reached sample size (171 controls, 329 cases, control : case ratio approx. 0.5) enabled to distinguish the difference in relative distribution of any entity (±13%) as statistically significant. Similarly, regarding the mean relative frequency as 50%, the study is able to detect difference (±13%) with 95% confidence.

### 3.2. SNPs and Haplotype Analysis

The studied polymorphisms +785C/T (rs2230054), +1208C/T (rs1126579), and +1440A/G (rs1126580) were in HWE in the control group (*P* > 0.05). No significant differences of all allele and genotype frequencies between the CP and control groups were found (see Table 2).

The distribution of genotype frequencies of all studied *CXCR2* gene variants was similar between men and women (data not shown). The SNPs in the *CXCR2* gene (+785C/T (rs2230054), +1208C/T (rs1126579), and +1440A/G (rs1126580)) were in very tight LD with each other to various degrees (|*D*′| = 0.72 − 0.94). Only the haplogenotype TCA/TTG was found more frequently in CP patients than in controls (0.0% vs. 2.4%, *P* < 0.05, *P*_corr_ > 0.05), but the number of subjects in both groups was very low. In our population, no other association between *CXCR2* haplotypes or haplogenotypes and CP was found (*P* > 0.05 for both, see Tables 3 and 4, respectively).

### 3.3. Microbial Analysis

There were no relationships between variability in the three studied *CXCR2* SNPs and the presence of seven periodontal bacteria in 162 CP patients, and only *A. actinomycetemcomitans* was marginally associated with *CXCR2* +785C/T SNP (*P* = 0.06). *A. actinomycetemcomitans* occurred more frequently in men positive for the C allele of the *CXCR2* +785C/T polymorphism (61.8% vs. 41.1%, *P* < 0.05; OR = 2.31, 95% CI = 1.03-5.20) and for the T allele of the *CXCR2* +1208C/T variant (61.8% vs. 38.9%, *P* < 0.05; OR = 2.54, 95% CI = 1.13-5.71). The presence of *P. micra* was marginally associated with the T allele of +1208 SNP for the group of male CP patients (49.0% vs. 27.3%, *P* = 0.05; OR = 2.56, 95% CI = 0.93-7.08; see Table 5). In contrast, there were no differences between the frequencies of *CXCR2* gene variants and the presence of periodontal bacteria in the group of CP women (*P* > 0.05, data not shown).

## 4. Discussion

Periodontal disease is characterized by inflammatory processes of tissues surrounding the teeth in response to bacterial stimulation. This inflammatory process is responsible for the progressive loss of the collagen attachment of the tooth to the alveolar bone, leading to bone loss [29]. According to the statistical report by the Ministry of Health of the Czech Republic, 15-20% of the Czech population aged 35-44 years suffered from periodontal disease in 2014 [30].

Chemokines and their receptors play important roles in immunological responses, and thus their genetic contribution to various human inflammatory disorders needs investigation [31]. Several reports have suggested that the *CXCR2* variants might influence the susceptibility to chronic inflammatory conditions, especially rheumatoid and respiratory diseases [32–35]. The *CXCR2* gene variability has been associated with several disorders like systemic sclerosis and cryptogenic fibrosing alveolitis, with a strong linkage between the +785C, +1208T, and +1440G alleles [26]. The +785T allele has been found to be protective against chronic obstructive pulmonary disease [32]. In Slovakia, children with the SNP +1208T allele were significantly unrepresented in the recurrent acute pyelonephritis subgroup, and the carriage of the T allele (TT+CT genotypes vs. CC genotype) was linked with a reduced risk of developing this disease [36]. Moreover, analysis of SNP +1208 with serum levels of IL-8, its endogenous ligand, supports an interaction whereby the variant +1208T allele and high serum IL-8 confer synergistic protection against lung cancer [33].

In our research, we focused on 3 polymorphisms in the *CXCR2* receptor +785C/T, +1208T/C, and +1440G/A, which were previously investigated in Brazilian patients with CP [21]. We detected similar allele, genotype, and haplotype frequencies of the all studied *CXCR2* gene polymorphisms between CP patients and controls (*P* > 0.05). In contrast, the +1440GG genotype, originally described by Viana et al. [37], was suggested as a protective factor against CP in Brazilians [21]. The differences between the results in the Czech and Brazilian study [21] could be caused by the interpopulation variability. The *CXCR2* +1440 minor allele frequencies were found to be 46.5% in Czech healthy nonperiodontitis controls vs. 57.4% in Brazilian controls [21]. However, our result is in line with the minor allele frequency (43.3%) in the European population [38].

If we arranged haplotypes as genotypes, the carriers of the TCA/TTG (or TTG/TCA) variant seemed to be more susceptible to CP development (*P* < 0.05). On the other hand, the number of carriers of this haplogenotype is too small to demonstrate any significant association with CP after correction for multiple comparisons. Viana et al. found that patients carrying the haplotypes TCA and CCG were more predetermined to CP development, whereas CCA and TCG haplotypes seemed to be protective against CP. In addition, white nonsmoking patients carrying the CTG/TCA variant were more likely to develop periodontal disease, whereas CTG/TCG patients seemed to be protected [21]. Our result matches with a comparable finding elsewhere: TTG/TCA and CTG/TCA haplotypes were associated with CP risk in two different populations, i.e., the Czech and Brazilian [21]. In addition, no CTG/TCG haplotype carrier was present in our population.

A lot of studies have focused on the relationship between the selected gene variants and the presence of periodontal bacteria studied in the recent review and meta-analysis by Nibali et al. [39]. Evidence suggests that genetic factors can influence periodontitis risk, modulating disease elements such as the susceptibility to microbial colonization and the nature of subsequent host-microbe interaction [40, 41]. Our previous research into *IL-8* gene polymorphisms in CP and aggressive periodontitis (AgP) patients shows the association between *IL-8* genotypes and the occurrence of specific periodontal bacteria [24]. In our earlier study, we reported significant differences in the colonization of the oral cavity with *P. gingivalis* (70.5% in CP patients vs. 28% in controls), *T. forsythia* (92.3% in CP patients vs. 56.3% in controls), and *P. micra* (87.2% in CP patients vs. 56.3% in controls) between CP patients and healthy controls [28]. We also determined that *IL-17A* -197A/G (rs2275913) polymorphism was associated with the presence of *T. forsythia* and *T. denticola* in CP patients [27] and that *IL-4* gene polymorphisms in CP patients could predispose to altered cytokine production after bacterial stimulation [42].

Although *A. actinomycetemcomitans* is more often associated with AgP than with CP, Gaetti-Jardim et al. [43] detected the bacteria by the PCR method in 44% of CP patients. In the Brazilian study, the *IL-4* haplotypes, but not the *IL-8* haplotypes, were associated with the presence of *A. actinomycetemcomitans* in CP patients [41]. Nibali et al. reported an association between the variability in the *IL-6* gene and *A. actinomycetemcomitans* in CP patients. The strong association of *IL-6* -174 GG homozygotes with the presence of *A. actinomycetemcomitans* in all subjects and in the subgroup of only white subjects was observed [42]. In our preliminary study, no significant association between the *CXCR2* +1208C/T (rs1126579) SNP and IL-8 plasma levels and the occurrence of the selected periodontal bacteria in 41 CP patients was found [24]. This result was confirmed in the present study on a larger sample size (*N* = 162). After the gender stratification, the presence of *A. actinomycetemcomitans* was significantly associated with *CXCR2* +785C/T and *CXCR2* +1208C/T SNPs, but only in Czech men.

## 5. Conclusions

This study did not confirm any significant association between the investigated SNPs in the *CXCR2* gene and chronic periodontitis. However, the *CXCR2* gene variants can be associated with subgingival colonization of the selected G- bacteria in men with CP in the Czech population.

## Figures and Tables

**Table 1:** Demographic data of CP patients and healthy nonperiodontitis controls.

Characteristics	Controls (*N* = 171)	CP (*N* = 329)
Age (mean ± SD, years)	47.56 ± 11.80	54.03 ± 8.99^∗^
Gender (males/females)	82/89	150/179
Smoking (no/yes) (%)	73.68/26.32	72.49/27.18
BMI (mean ± SD, kg m^−2^)	25.45 ± 3.61	26.20 ± 3.77
PD (mean ± SD, mm)	1.21 ± 0.24	3.26 ± 0.81^∗^
AL (mean ± SD, mm)	1.33 ± 0.21	3.94 ± 1.05^∗^
PI (mean ± SD, mm)	0.34 ± 0.14	0.83 ± 0.49^∗^
GI (mean ± SD, mm)	0.38 ± 0.31	0.81 ± 0.36^∗^

AL = attachment loss; CP = chronic periodontitis; GI = gingival index; *N* = number of subjects; PD = probing depth; PI = plaque index; SD = standard deviation. ^∗^*P* < 0.05.

**Table 2:** *CXCR2* genotype and allele frequencies in CP patients and healthy nonperiodontitis controls.

Genotypes	Controls	CP	*P* value	OR (95% CI)
Alleles	*N* = 171 (%)	*N* = 329 (%)		
*CXCR2 +785*				
CC	41 (24.0)	81 (24.6)	—	1.00
CT	93 (54.4)	166 (50.5)	0.37	0.90 (0.57-1.42)
TT	37 (21.6)	82 (24.9)	0.39	1.12 (0.65-1.93)
C allele	175 (51.2)	328 (49.8)	—	1.00
T allele	167 (48.8)	330 (50.2)	0.37	1.05 (0.81-1.37)

*CXCR2 +1208*				
CC	45 (26.3)	91 (27.7)	—	1.00
CT	90 (52.6)	174 (52.9)	0.47	0.96 (0.62-1.48)
TT	36 (21.1)	64 (19.5)	0.37	0.88 (0.51-1.51)
C allele	180 (52.6)	356 (54.1)	—	1.00
T allele	162 (47.4)	302 (45.9)	0.35	0.94 (0.73-1.22)

*CXCR2 +1440*				
AA	35 (20.5)	59 (17.9)	—	1.00
AG	89 (52.0)	171 (52.0)	0.34	1.14 (0.70-1.86)
GG	47 (27.5)	99 (30.1)	0.25	1.25 (0.73-2.15)
A allele	159 (46.5)	289 (43.9)	—	1.00
G allele	183 (53.5)	369 (56.1)	0.24	1.11 (0.85-1.44)

CI = confidential interval; CP = chronic periodontitis; *N* = number of subjects; OR = odds ratio.

**Table 3:** Estimated frequencies (%) of *CXCR2* haplotypes in CP patients and healthy nonperiodontitis controls.

*CXCR2* +785C/T	*CXCR2* +1208C/T	*CXCR2* +1440A/G	Controls (*N* = 171)	CP (*N* = 329)	*P* value	OR (95% CI)
C	T	G	42.3	41.4	0.79	0.96 (0.74-1.26)
T	C	A	39.6	37.9	0.62	0.94 (0.72-1.22)
T	C	G	5.8	8.3	0.14	1.49 (0.87-2.56)
C	C	A	4.7	5.3	0.73	1.11 (0.60-2.09)
T	T	G	2.8	3.7	0.53	1.28 (0.58-2.81)
C	C	G	2.5	2.6	0.92	1.04 (0.46-2.34)
C	T	A	1.6	0.6	0.10	0.31 (0.07-1.30)
T	T	A	0.6	0.3	0.52	0.52 (0.07-3.70)

CI = confidential interval; CP = chronic periodontitis; *N* = number of subjects; OR = odds ratio.

**Table 4:** Distribution of *CXCR2* haplotypes (arranged as genotypes) in CP patients and healthy nonperiodontitis controls.

Haplogenotypes	Controls	CP	*P* value	OR (95% CI)
+785 +1208 +1440/+785 +1208 +1440	*N* = 171 (%)	*N* = 329 (%)		
CTG/CTG	27 (15.8)	52 (15.8)	0.55	1.00 (0.60-1.66)
CTG/TCA	67 (39.2)	128 (38.6)	0.51	0.99 (0.68-1.44)
CTG/TCG	6 (3.5)	12 (3.6)	0.58	1.04 (0.38-2.82)
CTG/CCA	5 (2.9)	11 (3.3)	0.52	1.15 (0.39-3.36)
CTG/TTG	6 (3.5)	5 (1.5)	0.13	0.42 (0.13-1.41)
CTG/CCG	6 (3.5)	11 (3.3)	0.55	0.95 (0.35-2.62)
CTG/CTA	2 (1.2)	2 (0.6)	0.42	0.52 (0.07-3.70)
CTG/TTA	0 (0.0)	2 (0.6)	0.43	^#^
TCA/TCA	21 (12.3)	42 (12.8)	0.50	1.05 (0.60-1.83)
TCA/TCG	12 (7.0)	21 (6.4)	0.46	0.90 (0.43-1.88)
TCA/CCA	9 (5.3)	13 (4.0)	0.32	0.74 (0.31-1.77)
TCA/TTG	0 (0.0)	8 (2.4)	0.03^∗^	^#^
TCA/CCG	3 (1.8)	5 (1.5)	0.55	0.86 (0.20-3.66)
TCA/CTA	2 (1.2)	0 (0.0)	0.12	^#^
TCA/TTA	2 (1.2)	0 (0.0)	0.12	^#^
TCG/TCG	0 (0.0)	5 (1.5)	0.12	^#^
TCG/TTG	1 (0.6)	3 (0.9)	0.58	1.56 (0.16-15.15)
TCG/CCG	0 (0.0)	1 (0.3)	0.66	^#^
CCA/CCA	0 (0.0)	3 (0.9)	0.28	^#^
CCA/CCG	0 (0.0)	1 (0.3)	0.95	^#^
CCA/CTA	1 (0.6)	1 (0.3)	0.57	0.52 (0.03-8.34)
TTG/TTG	1 (0.6)	3 (0.9)	0.58	1.56 (0.16-15.15)

CI = confidential interval; CP = chronic periodontitis; *N* = number of subjects; OR = odds ratio. ^∗^*P* < 0.05 by the Fisher exact test (without correction for multiple comparisons), but there is a low *N* in both groups. ^#^OR not calculated because of the presence of zero.

**Table 5:** *CXCR2* gene variants and the presence of periodontal bacteria in 62 male CP patients.

Allele frequencies (%)		*CXCR2* +785		*CXCR2* +1208		*CXCR2* +1440	
		C	T	C	T	A	G
*A. actinomycetemcomitans*	Neg. *N* = 90	41.1	58.9	61.1	38.9	45.6	54.4
	Pos. *N* = 34	61.8^∗^	38.2	38.2	61.8^∗^	29.4	70.6

*T. forsythia*	Neg. *N* = 14	35.7	64.3	50.0	50.0	35.7	64.3
	Pos. *N* = 110	48.2	51.8	55.5	44.5	41.8	58.2

*P. gingivalis*	Neg. *N* = 38	47.4	52.6	55.3	44.7	36.8	63.2
	Pos. *N* = 86	46.5	53.5	54.7	45.3	43.0	57.0

*T. denticola*	Neg. *N* = 40	52.5	47.5	47.5	52.5	40.0	60.0
	Pos. *N* = 84	44.0	56.0	58.3	41.7	41.7	58.3

*P. micra*	Neg. *N* = 22	31.8	68.2	72.7	27.3	40.9	59.1
	Pos. *N* = 102	50.0	50.0	51.0	49.0^∗^	41.2	58.8

*P. intermedia*	Neg. *N* = 56	50.0	50.0	53.6	46.4	35.7	64.3
	Pos. *N* = 68	44.1	55.9	55.9	44.1	45.6	54.4

*F. nucleatum*	Neg. *N* = 2	0.0	100.0	100.0	0.0	0.0	100.0
	Pos. *N* = 122	47.5	52.5	54.1	45.9	41.8	58.2

*N* = number of alleles; Neg. = negative; Pos. = positive. ^∗^*P* ≤ 0.05.

## Data Availability

The clinical and genetic data used to support the findings of this study are restricted by the Committees for Ethics of the Faculty of Medicine, Masaryk University, Brno (no. 13/2013), in order to protect patient privacy. Data are available from Lydie Izakovicova Holla (holla@med.muni.cz) for researchers who meet the criteria for access to confidential data.
